# Incidence and risk of sepsis following appendectomy: a nationwide population-based cohort study

**DOI:** 10.1038/s41598-020-66943-5

**Published:** 2020-06-23

**Authors:** Meng-Che Wu, Hsi-Kai Tsou, Cheng-Li Lin, James Cheng-Chung Wei

**Affiliations:** 1 Institute of Medicine, Chung Shan Medical University, Taichung, Taiwan; 2 Division of Gastroenterology, Children’s Medical Center, Taichung Veterans General Hospital, Taichung, Taiwan; 3 Functional Neurosurgery Division, Neurological Institute, Taichung Veterans General Hospital, Taichung, Taiwan; 4 Department of Rehabilitation, Jen-Teh Junior College of Medicine, Nursing and Management, Miaoli County, Taiwan; 5 College of Medicine, China Medical University, Taichung, Taiwan; 6 Management Office for Health Data, China Medical University Hospital, Taichung, Taiwan; 7 Department of Rheumatology, BenQ Medical Center, The Affiliated BenQ Hospital of Nanjing Medical University, Nanjing, China; 8 Beijing Tsinghua Changgung Hospital, School of Clinical Medicine, Tsinghua University, Beijing, China; 9 Division of Allergy, Immunology and Rheumatology, Chung Shan Medical University Hospital; Institute of Medicine, College of Medicine, Chung Shan Medical University, Taichung, Taiwan; 10 Graduate Institute of Integrated Medicine, China Medical University, Taichung, Taiwan

**Keywords:** Diseases, Gastroenterology, Medical research

## Abstract

Appendectomy is a frequently performed surgical procedure; however, long-term consequences have not been fully explored. We used a nationwide population-based cohort to determine whether patients undergoing appendectomy are at an increased risk of sepsis. Overall, 252,688 patients undergoing appendectomy and 252,472 matched controls were identified from the National Health Insurance Research Database in Taiwan. A propensity score analysis was used for matching age, sex, index year and comorbidities at a ratio of 1:1. Multiple Cox regression and stratified analyses were used to estimate the adjusted hazard ratio (aHR) of developing sepsis. Patients undergoing appendectomy had a 1.29 times (aHR: 1.29; 95% confidence interval [CI], 1.26–1.33) higher risk of developing sepsis than those not undergoing. Patients aged 20–49 years had a 1.58-fold higher risk of sepsis in the appendectomy cohort (aHR; 95% CI, 1.50–1.68). Also, having undergone appendectomy, patients had a higher likelihood of sepsis, regardless of sex and with or without comorbidities. Patients with <1 year follow-up showed a 1.98-fold risk of sepsis in the appendectomy cohort. Patients with 1–4 and ≥5 years follow-up showed a 1.29 and 1.11-fold risk of sepsis, respectively. Future research is required to elucidate the possible immuno-pathological mechanisms of these associations.

## Introduction

Sepsis is a clinical heterogeneous syndrome that is defined as a ‘life-threatening organ dysfunction caused by a dysregulated host response to an infection’^1,2^. Sepsis and the subsequent systemic inflammatory response can lead to multiple organ dysfunction and even death^1^. It remains a clinical challenge for clinicians and researchers.

Appendectomy is one of the most frequently performed emergent abdominal procedures worldwide. However, it has recently been recognised that the appendix is not just a vestigial structure of the gut, but rather is an important organ for the development and preservation of the gut immune system^3,4^. The human vermiform appendix provides a continuous source of commensal flora, thereby crowding out potentially unhealthy microorganisms, restoring diversity and stability of the gut microbiome and contributing to defensive immune mechanisms over a lifetime. Thus, it is known as a ‘safe house’ for normal gut flora. The appendix has been significantly associated with recurrent *Clostridioides difficile* infection^4^ and recurrence of small bowel bacterial overgrowth after antibiotics administration^5^. Studies have also examined the association between antecedent removal of the appendix and the risk of various diseases such as systemic lupus erythematosus^6^, rheumatoid arthritis^7^, inflammatory bowel disease^8^ and cancers^9–11^. These conditions reflect the important role of the appendix in microbial ecology and intestinal mucosal immunity.

The short-term complications of appendectomy are well-studied, whereas only limited long-term consequences have been assessed^12^. The absence of the appendix might alter immune function and the gut microbiome. It is postulated that in susceptible populations, the intestinal epithelium becomes hyper-permeable and apoptotic during critical illness, which results in intestinal flora leaking into the systemic circulation and potentially leading to systemic inflammation and organ failure. The gut microbiota is also thought to play a pivotal role in the pathogenesis of sepsis^13^. It has been shown that the gut microbiome composition of patients with sepsis is profoundly distorted^14^. Thus, we hypothesised that antecedent appendectomy could impact the future risk of sepsis and evaluated this hypothesis by analysing a nationwide population-based retrospective cohort from the Taiwanese National Health Insurance Research Database (NHIRD).

## Results

We identified 252,688 patients undergoing appendectomy and 252,472 matched controls between 2000 and 2013 from the National Health Insurance Research Database in Taiwan. The median follow-up periods for the appendectomy and non-appendectomy cohorts were 7.04 and 6.96 years, respectively. Table 1 shows the demographic characteristics of the patients. There were 71.2%, 16.9% and 11.9% of patients in the 20–49 age group, 50–64 age group and ≥65 age group, respectively, and about 48.4% of participants were women and about 51.6% of patients were men. The median age in the exposed cohort was 41.8 ± 16.8 years, and the average age in the unexposed cohort was 41.7 ± 17.0 years. In terms of comorbidities, there were no statistically significant differences between the exposed cohort and the unexposed cohort.

**Table 1:** Baseline characteristics for individuals with and without appendectomy.

	Appendectomy				P value
	No		Yes		
	N = 252,472		N = 252,688		
	n	%	n	%	
Age (years)					0.97
20 − 49	179,762	71.2	179,847	71.2	
50 − 64	42,590	16.9	42,644	16.9	
≥65	30,120	11.9	30,197	12.0	
Median (SD) ^∗^	41.7	17.0	41.8	16.8	0.01
Sex					0.97
Women	122,127	48.4	122,218	48.4	
Men	130,345	51.6	130,470	51.6	
Comorbidity					
Diabetes mellitus	12,884	5.10	12,991	5.14	0.54
Hypertension	24,341	9.64	24,456	9.68	0.65
Hyperlipidemia	5465	2.16	5558	2.20	0.40
Atrial fibrillation	1486	0.59	1580	0.63	0.09
Stroke	5671	2.25	5769	2.28	0.38
Congestive heart failure	2193	0.87	2290	0.91	0.15

Chi-square test; ^∗^Mann-Whitney U test; SD, standard deviation.

Table 2 displays the incidence and risk factors for sepsis. The incidence rate was 6.05 per 1,000 person-years among the appendectomy cohort. After adjustment, patients with appendectomy had a significantly higher risk of developing sepsis than those without appendectomy (aHR, 1.29; 95% CI, 1.26–1.33; P < 0.001). Further analysis of the extended Cox models with time-dependent terms showed similar results, indicating that the strength of the association increased over time (HR [P-value] for appendectomy, 1.23 [P < 0.001] and for the interaction term of appendectomy and time, 1.09 [P < 0.001]).

**Table 2:** The incidence of and risk factors for sepsis.

	Event	PY	Rate ^#^	Crude HR (95% CI)	Adjusted HR ^&^ (95% CI)
Appendectomy					
No	8678	1,808,705	4.80	1.00	1.00
Yes	10847	1,791,553	6.05	1.26(1.23, 1.30)***	1.29(1.26, 1.33)***
Age					
20 − 49	5005	2,694,333	1.86	1.00	1.00
50 − 64	4467	562,009	7.95	4.35(4.18, 4.53)***	3.59(1.25, 1.33)***
≥65	10053	343,916	29.2	16.2(15.7, 16.8)***	10.2(9.85, 10.6)***
Gender					
Women	8851	1,752,543	5.05	1.00	1.00
Men	10674	1,847,716	5.78	1.14(1.11, 1.18)***	1.15(1.12, 1.18)***
Comorbidity					
Diabetes mellitus					
No	15,364	3,460,921	4.44	1.00	1.00
Yes	4161	139,337	29.9	6.82(6.59, 7.06)***	2.01(1.94, 2.09)***
Hypertension					
No	12,917	3,343,842	3.86	1.00	1.00
Yes	6608	256,417	25.8	6.85(6.65, 7.06)***	1.48(1.42, 1.53)***
Hyperlipidemia					
No	18,147	3,538,639	5.13	1.00	1.00
Yes	1378	61,619	22.4	4.38(4.15, 4.63)***	1.05(0.99, 1.12)
Atrial fibrillation					
No	18,898	3,586,432	5.27	1.00	1.00
Yes	627	13827	45.4	8.64(7.97, 9.35)***	1.31(1.21, 1.43)***
Stroke					
No	17,221	3,541995	4.86	1.00	1.00
Yes	2304	58,263	39.5	8.22(7.87, 8.59)***	1.56(1.49, 1.64)***
Congestive heart failure					
No	18,492	3,581,114	5.16	1.00	1.00
Yes	1033	19,144	54.0	10.5(9.86, 11.2)***	1.68(1.57, 1.80)***

CI confidence interval; HR, hazard ratio; PY, person-years;

^#^Incidence rate per 1,000 person-years;

^&^Multivariable analysis including age, gender and comorbidities of diabetes mellitus, hypertension, hyperlipidemia, atrial fibrillation, stroke and congestive heart failure;

*** P < 0.001.

Older age groups were associated with a higher risk of developing sepsis when they were compared with the 20–49 age group (aHR, 3.59; 95% CI, 3.45–3.75; P < 0.001 in the 50–64 age group & aHR, 10.2; 95% CI, 9.85–10.6; P < 0.001 in the ≥65 age group). Compared with women, men had a higher risk of sepsis (aHR, 1.15; 95% CI, 1.12–1.18; P < 0.001). In terms of comorbidities, patients with diabetes mellitus (aHR, 2.01; 95% CI, 1.94–2.09; P < 0.001), hypertension (aHR, 1.48; 95% CI, 1.42–1.53; P < 0.001), atrial fibrillation (aHR, 1.31; 95% CI, 1.21–1.43; P < 0.001), stroke (aHR, 1.56; 95% CI, 1.49–1.64; P < 0.001) and congestive heart failure (aHR, 1.68; 95% CI, 1.57–1.80; P < 0.001), were all at higher risk of sepsis.

**Figure 1:**
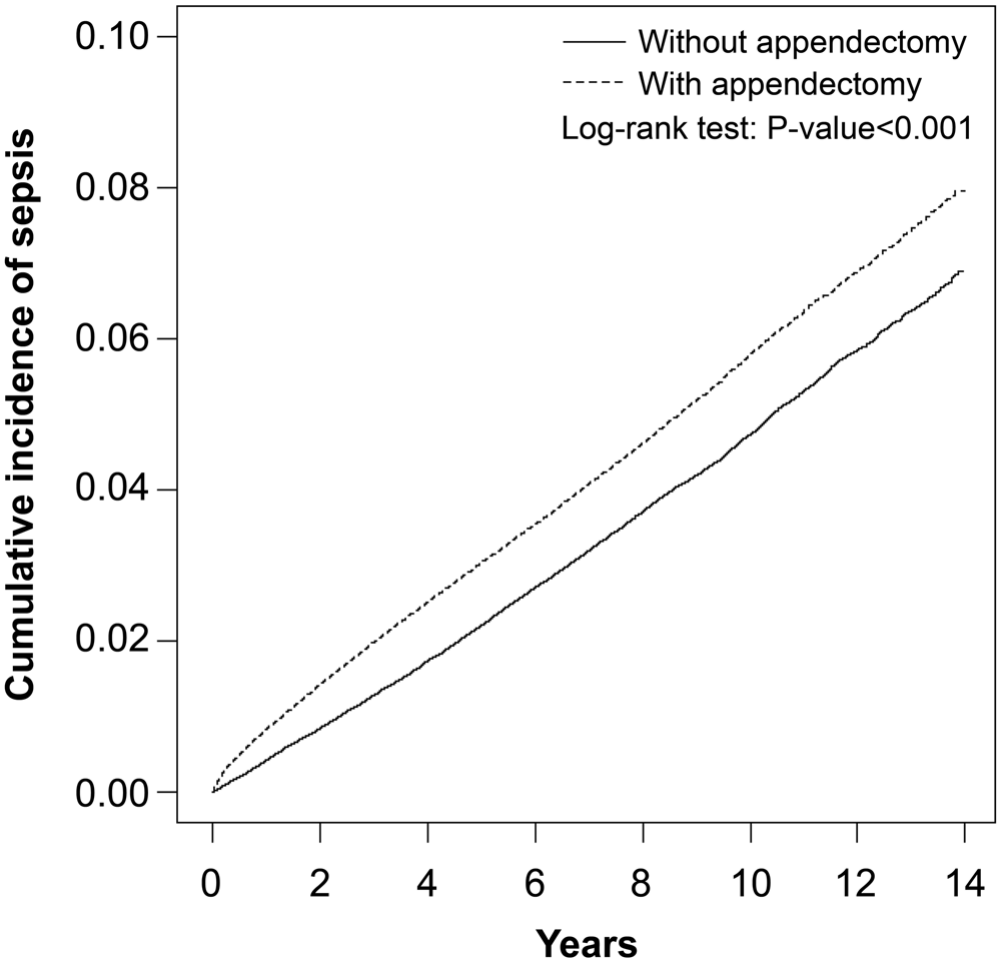
Cummulative incidence of sepsis between individuals with and without appendectomy.

In Table 3, stratified analyses were performed to assess the association between appendectomy and sepsis based on demographic characteristics. In patients aged 20–49 years, compared with the unexposed cohort, there was a 1.58-fold higher risk of sepsis in the exposed cohort (aHR; 95% CI, 1.50–1.68; P < 0.001). In patients aged 50–64 years, compared with the unexposed cohort, there was 1.40-fold higher risk of sepsis in the exposed cohort (aHR; 95% CI, 1.32–1.48; P < 0.001). In patients aged ≥65 years, compared with the unexposed cohort, there was a 1.13-fold higher risk of sepsis in the exposed cohort (aHR; 95% CI, 1.09–1.18; P < 0.001). Among women, compared with patients without appendectomy, there was a 1.39-fold higher risk of sepsis in patients with appendectomy (aHR; 95% CI, 1.33–1.45; P < 0.001). Among men, compared with patients without appendectomy, there was 1.22-fold higher risk of sepsis in patients with appendectomy (aHR; 95% CI, 1.17–1.27; P < 0.001). For patients without any comorbidity, compared with the unexposed cohort, there was 1.51-fold higher risk of sepsis in the exposed cohort (aHR; 95% CI, 1.45–1.57; P < 0.001). For patients with any comorbidity, compared with the unexposed cohort, there was a 1.10-fold higher risk of sepsis in the exposed cohort (aHR; 95% CI, 1.06–1.15; P < 0.001). For patients whose years of follow-up were <1 year, there was a 1.98-fold higher risk of sepsis in the exposed cohort when compared with the unexposed cohort (aHR; 95% CI, 1.84–2.14; P < 0.001). For patients whose years of follow-up were 1–4 years, there was a 1.29-fold higher risk of sepsis in the exposed cohort when compared with the unexposed cohort (aHR; 95% CI, 1.23–1.34; P < 0.001). For patients whose years of follow-up were ≥5 years, there was a 1.11-fold higher risk of sepsis in the exposed cohort when compared with the unexposed cohort (aHR; 95% CI, 1.06–1.16; P < 0.01). In Table 4, similar results were observed for sepsis through inverse probability of treatment weights propensity score methods; the appendectomy cohort had a higher risk of sepsis than the non-appendectomy cohort. We further excluded individuals with a follow-up period of <3 months and analysed only new sepsis occurring at least 90 days after appendectomy. Patients with appendectomy had a significantly higher risk of developing sepsis than those without appendectomy (aHR, 1.86; 95% CI, 1.61–2.14; P < 0.001). The Kaplan–Meier curves are shown in Fig. 1. The cumulative incidence of sepsis was lower in patients without appendectomy than in patients with appendectomy.

**Table 3:** Incidence and hazard ratio of sepsis for individuals with and without appendectomy.

	Appendectomy						Crude HR (95% CI)	Adjusted HR ^&^ (95% CI)
	No			Yes				
	Event	PY	Rate ^#^	Event	PY	Rate ^#^		
Age								
20 − 49	1925	1,345,408	1.43	3080	1,348,925	2.28	1.60(1.51, 1.69)***	1.58(1.50, 1.68)***
50 − 64	1905	28,6471	6.65	2562	275538	9.30	1.40(1.32, 1.49)***	1.40(1.32, 1.48)***
≥65	4848	176,826	27.4	5205	167,090	31.2	1.14(1.10, 1.19)***	1.13(1.09, 1.18)***
Gender								
Women	3775	879,724	4.29	5076	872,819	5.82	1.36(1.30, 1.41)***	1.39(1.33, 1.45)***
Men	4903	928,981	5.28	5771	918,734	6.28	1.19(1.15, 1.24)***	1.22(1.17, 1.27)***
Comorbidity ^§^								
No	4258	1,617,489	2.63	6137	1,605,213	3.82	1.45(1.40, 1.51)***	1.51(1.45, 1.57)***
Yes	4420	191,216	23.1	4710	186,340	25.3	1.09(1.05, 1.14)***	1.10(1.06, 1.15)***
Follow-up years								
<1	1055	249,563	4.23	2073	248,222	8.35	1.97(1.83, 2.13)***	1.98(1.84, 2.14)***
1–4	3682	820,155	4.49	4606	813,688	5.66	1.26(1.21, 1.32)***	1.29(1.23, 1.34)***
≥5	3941	738,986	5.33	4168	729,643	5.71	1.07(1.03, 1.12)**	1.11(1.06, 1.16)**

CI confidence interval; HR, hazard ratio; PY, person-years;

^#^Incidence rate per 1,000 person-years;

^&^Multivariable analysis including age, gender and comorbidities of diabetes mellitus, hypertension, hyperlipidemia, atrial fibrillation, stroke and congestive heart failure;

^§^Individuals with any comorbidity of diabetes mellitus, hypertension, hyperlipidemia, atrial fibrillation, stroke and congestive heart failure were classified into the comorbidity group;

**P < 0.01, ***P < 0.001.

**Table 4:** Sensitivity analyses of sepsis risk for individuals with and without appendectomy using inverse probability of treatment weights propensity score methods and individuals excluded due to a follow-up period of <3 months.

Variable	Crude HR (95% CI)	Adjusted HR ^&^ (95% CI)
Inverse probability of treatment weights	1.26(1.24, 1.29)***	1.31(1.28, 1.33)***
Excluded due to a follow-up period of <3 months	2.03(1.76, 2.33)***	1.86(1.61, 2.14)***

CI, confidence interval; HR, hazard ratio; PY, person-years;

^&^Multivariable analysis including age, gender and comorbidities of diabetes mellitus, hypertension, hyperlipidemia, atrial fibrillation, stroke and congestive heart failure;

***P < 0.001.

## Discussion

In this large, population-based cohort study, we observed a 1.29-fold higher risk of sepsis in patients undergoing appendectomy, and it was especially higher in patients younger than 50 years (aHR: 1.58, 95% CI, 1.50–1.68) and those within five years of follow-up after appendectomy. Diabetes was a comorbidity with a relatively higher adjusted hazard ratio (aHR: 2.01, 95% CI, 1.94–2.09) for sepsis compared with other comorbidities. Furthermore, the cumulative incidence of sepsis among patients undergoing appendectomy significantly increased with time. These findings support our hypothesis that there is an association between prior appendectomy and the future risk of sepsis. To the best of our knowledge, this is the first and largest epidemiological study to use a nationwide longitudinal dataset to identify an increased sepsis risk among patients with appendectomy. These results highlight the possibility that the absence of a vermiform appendix might provoke an increased risk of sepsis.

The pathophysiology underlying the relationship between an appendectomy and subsequent sepsis remains uncertain. The appendix is the primary site of production of secretory immunoglobulin A^15^, which binds to pathogenic bacteria with high affinity and promotes their elimination^16^. Nevertheless, it also binds to the commensal gut flora with low affinity and plays a crucial role in host protection. Therefore, the appendix plays an important role in regulating the size and composition of the gut microbiota^16^. The function of the appendix is to act as a reservoir of commensal flora to rapidly re-inoculate the gut through biofilm regeneration and shedding following enteric infections or antibiotics administration^16–18^. We speculate that the lack of an appendix could influence the restoration of the gut microbiome, which could increase susceptibility to sepsis and related organ dysfunction. The gut has been considered a motor of sepsis and multiple organ dysfunction syndrome for the past one third century^19^. It is hypothesised that the intestinal epithelium becomes highly permeable and apoptotic during critical illness, which results in intestinal flora leaking into the systemic circulation and potentially leading to systemic inflammation and organ failure. Also, various pre-clinical studies have revealed the existence of so-called ‘gut-organ axes’, such as the gut-brain axis and the gut-lung axis^14^. In addition to cytokines, communication on these axes might be mediated by microbe-associated molecular patterns, such as peptidoglycan, LPS, flagellin and microbiome-derived metabolites, which can be transferred from the gut to the systemic circulation^14^. These microbe-associated molecular patterns have the potential to modulate immune cells to enhance the systemic inflammatory response. The appendix also has the highest concentration of mucosa-associated lymphoid tissue and is capable of responding to various pathogens and microbial antigens present in the gut. Removal of the appendix, which is an immune organ and a reservoir of beneficial flora, might lead to local and distant insults through changes in defence mechanisms and disruption of the equilibrium regulated by complex crosstalk between the gut, mucosal immunity and the microbiota. This process could lead to the progression and pathogenesis of sepsis and organ dysfunction in susceptible populations. For instance, cancer patients who must receive cytotoxic chemotherapies and subsequently suffer intestinal mucosal disturbance, are particularly at risk of sepsis.

The second finding of this study was that appendectomy led to a significantly higher risk of sepsis in the middle aged (20–49 years) patients (aHR 1.58, 95% CI, 1.50–1.68) than in those with prior appendectomy at >65 years (aHR 1.13, 95% CI, 1.09, 1.18). In elderly individuals, the function of the appendix might be attenuated, given the length/diameter and the number of lymphoid follicles of the human vermiform appendix degenerate with age^20^. Therefore, appendectomy might be less likely to affect their immune defences. Another finding of our study was that diabetes was a comorbidity with a relatively higher adjusted hazard ratio (aHR: 2.01, 95% CI, 1.94–2.09) compared with other comorbidities. We speculate that appendectomy may worsen pre-existing dysbiosis in patients with diabetes mellitus and thus increased the risk of sepsis.

In addition, sepsis might be induced by complications of recent appendicitis and subsequent surgical procedure, such as intra-abdominal abscess, surgical site infection and intestinal obstruction. We also found that there was a relatively higher risk of sepsis (aHR: 1.98, 95% CI, 1.84–2.14) within the first years of follow-up after undergoing appendectomy. However, the risk of sepsis was still significantly higher in the follow-up periods of 1–4 and ≥5 years. One recent study by Ninh *et al*.^21^ reported that 311 patients (0.43%) among 72,538 participants developed post-appendectomy related sepsis, and open appendectomy increased the risk of sepsis. We further analysed only new sepsis occurring at least 90 days after appendectomy and found that patients with appendectomy had a significantly higher future risk of sepsis than those without appendectomy. There is increasing evidence to show that non-surgical management with antibiotics might be an effective and safe treatment for acute uncomplicated appendicitis in children and adults^17,22,23^. Our study provides evidence of a correlation between previous appendectomy and subsequent sepsis risk. Therefore, we recommend thoughtful consideration before performing incidental or prophylactic appendectomy. In other words, the potential benefit of appendectomy must be weighed judiciously after considering the patient’s personal risk-benefit profile. In addition, probiotics that can modulate the function of the intestinal immune system might have a beneficial impact on those undergoing appendectomy; further dedicated research on this issue is warranted.

The major strengths of this study were the large sample size and the relatively long duration of the follow-up, in which a complete history of medical service use was available for all cases and controls. Therefore, our study design minimised selection, information and recall bias, which made testing our hypothesis feasible. Furthermore, we used strict exclusion criteria and propensity score matching to control for potential confounders. Nonetheless, there are several limitations to be noted. First, the NHIRD does not disclose information regarding the patients’ diet, socioeconomic status, family history, personal lifestyle, psychologic factors, body mass index and microbiomes of the study populations, which might be associated risk factors for the development of sepsis. Although we adjusted for various comorbidities and matched propensity scores, these unmeasured confounding factors might have affected our results. Second, accurately quantifying the incidence of sepsis is difficult. Because there is no definitive tissue or serological test for sepsis, the gold standard for sepsis diagnosis is clinical identification of life-threatening organ dysfunction caused by infection^2^. At the nationwide population level, this would require either a prospective cohort study or retrospective medical record review, the scale of which is unrealistic for routine disease surveillance. Therefore, routinely collected data are analysed to estimate the incidence of sepsis, mainly based on ICD coding of cases. However, sepsis patients comprise heterogeneous groups and are categorised with different diagnostic administrative codes. We focused on patients with diagnoses of sepsis (ICD-9-CM code 038.x, 003.1 and 036.2). The definition of sepsis was based on ICD-9-CM codes, rather than clinical diagnostic criteria such as the sepsis-3 definition^2^. This is an inherent limitation of population-based datasets such as the NHIRD. Although the accuracy of these codes has been validated^24–31^, Valentine *et al*. reported that the sensitivity of coding data regarding sepsis was 78% and 64% of intensive care unit (ICU) and non-ICU cases using ICD-10 codes at admission level^32^. Another study by Fleischmann-Struzek *et al*. also indicated that explicit sepsis coding strategies may underestimate sepsis incidence and implicit strategies (an infection code plus a code indicting organ dysfunction) might overestimate the incidence of sepsis^33^. Thus, potential misclassification is worthy of attention; however, we believe that any misclassification is non-differential for exposed and unexposed cohorts and that the undetected presence of an infectious code plus organ dysfunction would be more likely to lead to underestimation of the associations examined. Moreover, clinical judgement might vary between physicians, so diagnoses could also vary and affect their validity. However, the NHI in Taiwan has established an ad hoc committee to monitor the accuracy of the claim data to prevent violations. Third, due to the potential for residual confounding inherent in database research, the results should be interpreted with caution. Randomised controlled trials to demonstrate the effect of appendectomy and subsequent sepsis are laborious and resource intensive. Such a study is difficult to conduct due to ethical issues in randomising patients to undergo appendectomy to observe adverse outcomes. Finally, it remains uncertain whether the finding in our study can be extrapolated to other ethnic groups, as the majority of our patients were Chinese. Clinical studies should be conducted in patients from other countries and must include people of different ethnicities to further elucidate the associations.

## Conclusion

Patients requiring appendectomy had a 1.29 times greater risk of developing sepsis than those not undergoing appendectomy. The cumulative incidence of sepsis among patients receiving appendectomy significantly increased over time. Future basic and clinic research is needed to clarify the pathogeneses underlying these associations.

## Methods

### Data source

This study employed the NHIRD, which consists of claim data after removing identifying information. The database was established in 1995 and includes records from over 99% of insured people who have resided in Taiwan. Diagnostic codes were defined based on the International Classification of Diseases, 9^th^ Revision, Clinical Modification (ICD-9-CM) codes. The Institutional Review Board of Research Ethics Committee II of China Medical University and Hospital [Approval number: CMUH104-REC2–115(CR-4)] has approved this study. Written consent from study subjects was not required and waived by the Institutional Review Board of Research Ethics Committee II of China Medical University and Hospital, because the NHIRD comprises de-identified data for research purposes. The study carried out in accordance with principles of Declaration of Helsinki.

### Study population

Patients aged >20 years who underwent appendectomy (ICD-9-CM Procedure Code: 47.0 and 47.1) were assigned to the case cohort. The index date was the date patients underwent the appendectomy. Participants who did not undergo appendectomy were assigned to the comparison cohort. The study excluded patients with a history of sepsis (ICD-9-CM: 003.1, 036.2 and 038.x) before the index date, those who underwent appendectomy before the year 2000 and those who lacked complete information in the NHIRD. The exposed cohort and the unexposed cohort were matched using propensity score matching at a 1:1 ratio by the index year, sex, age and comorbidities. The main outcome was a new-onset hospitalised diagnosis of sepsis (ICD-9-CM: 003.1, 036.2 and 038.x). All participants were observed until the end of 2013 or until their records were censored for death, emigration or discontinuation of enrolment in the NHIRD.

### Comorbidities

The baseline history of comorbidity comprised diabetes mellitus (ICD-9-CM: 250 and A181), hypertension (ICD-9-CM: 401–405, A260 and A269), hyperlipidemia (ICD-9-CM: 272 and A182), atrial fibrillation (ICD-9-CM: 427.31), stroke (ICD-9-CM: 430–438 and A290-A299) and congestive heart failure (ICD-9-CM: 428).

### Statistical analysis

The distributions of categorical demographic variables and comorbidities were compared between the exposed cohort and the unexposed cohort and the differences were examined using chi-squared tests. The median age of both cohorts was measured and tested using Mann-Whitney U test. The proportional hazards model assumption was also examined using a test of scaled Schoenfeld residuals. In the model evaluating the sepsis risk throughout the overall follow-up period, the results of the test revealed a significant relationship between the Schoenfeld residuals for appendectomy and follow-up time, suggesting that the proportionality assumption was violated (P < 0.001). Cox proportional hazard models were employed to assess the risk of developing sepsis associated with appendectomy after adjusting for the following covariates: age, gender and comorbidities of diabetes mellitus, hypertension, hyperlipidemia, atrial fibrillation, stroke and congestive heart failure. HRs with 95% confidence intervals (95% CIs) were calculated using this model.

Sub-analyses stratified by sex, age group, comorbidity and years of follow-up were also performed to assess the association between appendectomy and the subsequent risk of sepsis. Cumulative incidence rates and curves of sepsis were estimated and plotted by the Kaplan–Meier method, and log-rank tests were used to compare differences in time-to-event distributions between the case cohort and the comparison cohort. The two-tailed significance levels of all tests were set at 0.05. All data were analysed using SAS 9.4 software (SAS Institute Inc., Cary, NC, USA) and cumulative incidence curves were plotted in R software.

## Data Availability

The dataset used in this study is held by the Taiwan Ministry of Health and Welfare (MOHW). The Ministry of Health and Welfare must approve our application to access this data, and patient consent is exempted due to de-identification of the NHIRD (Database NHIR, Taiwan). Available online: http://nhird.nhri.org.tw/en/index.htm. All methods were performed in accordance with the relevant guidelines and regulations. Any researcher interested in accessing this dataset can submit an application form to the Ministry of Health and Welfare requesting access. Please contact the staff of MOHW (Email: stcarolwu@mohw.gov.tw) for further assistance. Taiwan Ministry of Health and Welfare Address: No.488, Sec. 6, Zhongxiao E. Rd., Nangang Dist., Taipei City 115, Taiwan (R.O.C.). Phone: +886-2-8590-6848. All relevant data are within the paper.
